# Targeting Metabolic Reprogramming in Acute Myeloid Leukemia

**DOI:** 10.3390/cells8090967

**Published:** 2019-08-24

**Authors:** Isabel Castro, Belém Sampaio-Marques, Paula Ludovico

**Affiliations:** 1 Life and Health Sciences Research Institute (ICVS), School of Medicine, University of Minho, 4710-057 Braga, Portugal; 2 ICVS/3B’s-PT Government Associate Laboratory, 4806-909 Braga/Guimarães, Portugal

**Keywords:** metabolism, acute myeloid leukemia, preclinical and clinical trials on metabolic targets

## Abstract

The cancer metabolic reprogramming allows the maintenance of tumor proliferation, expansion and survival by altering key bioenergetics, biosynthetic and redox functions to meet the higher demands of tumor cells. In addition, several metabolites are also needed to perform signaling functions that further promote tumor growth and progression. These metabolic alterations have been exploited in different cancers, including acute myeloid leukemia, as novel therapeutic strategies both in preclinical models and clinical trials. Here, we review the complexity of acute myeloid leukemia (AML) metabolism and discuss how therapies targeting different aspects of cellular metabolism have demonstrated efficacy and how they provide a therapeutic window that should be explored to target the metabolic requirements of AML cells.

## 1. Introduction

Acute myeloid leukemia (AML) is the most frequent acute leukemia in adults, with a median age at diagnosis of around 68 years. It is an aggressive disease, requiring an urgent accurate diagnosis and a multifaceted, timely, and effective therapeutic approach in order to achieve disease remission and even long-term cure [1]. Despite the progress in prognostic markers, the optimization of cytotoxic therapy and advances in supportive care, the long-term outcome of AML is not encouraging, particularly in elderly patients [2]. The intrinsic refractoriness of the disease in the eldest population in association with existing comorbidities contribute towards a high toxicity profile upon application of the standard cytotoxic therapy, yielding an overall survival (OS) lower than 1 year [3]. In fact, the genetic landscape of patients older than 60 years is different from the young ones, namely presenting adverse cytogenetic profile, overexpression of drug resistance genes, and a high frequency of mutations in genes involved in epigenetic regulation [4,5]. Noteworthy, mutations on these epigenetics-related genes are also documented in healthy elderly population in an age dependent manner, increasing the risk of hematologic neoplasms [6]. Thus, it is important for the old/refractory or unfit patients to look for more effective and less toxic therapies and, in such context, metabolic reprogramming occurring in leukemic cells presents as a promising target [7].

The strategies used by malignant cells to escape the mechanisms of homeostasis control are appreciably varied, with metabolic reprogramming playing a key role in the final outcome. Metabolic reprogramming is considered a hallmark of cancer and, basically, consists of providing a rewired metabolism to support high metabolic demands for the elevated rate of growth and proliferation. Indeed, to maintain tumor proliferation, expansion, and survival, a reprogramming of metabolism must be performed to satisfy key bioenergetics, biosynthetic and redox functions of malignant cells. In addition to the altered metabolism, several metabolites have a signaling function, promoting tumor growth and progression [8]. Numerous studies and clinical trials demonstrate both the importance of metabolic adaptation for the survival of AML cells and the therapeutic potential of attacking different metabolic pathways. In the last years, several drugs have been developed to target specific metabolic pathways and enzymes, metabolites, and signaling pathways. Several studies show an acceptable toxicity of some of these metabolism-affecting drugs even when used in combination with standard chemotherapy. In this review, we intend to highlight the most recent data in the field and, whenever possible, discuss the clinical impact.

## 2. Metabolic Reprogramming in Acute Myeloid Leukemia

**Figure 1:**
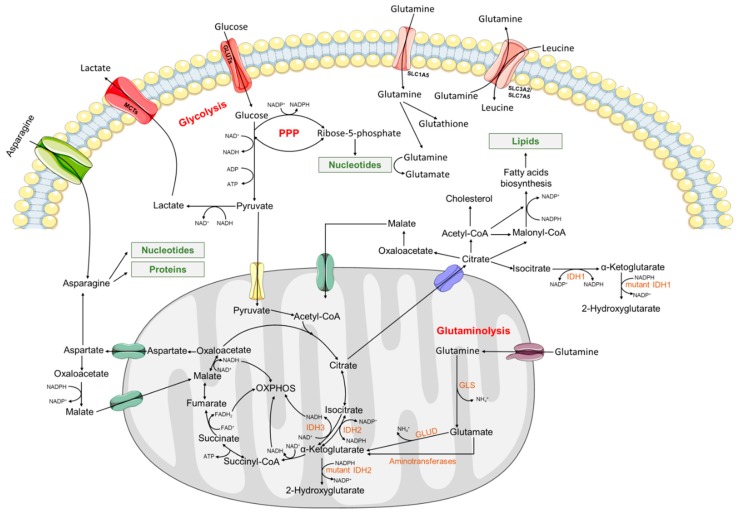
Overview of an acute myeloid leukemia cell’s metabolism. Metabolic reprogramming provides ATP and intermediates for the synthesis of nucleotides, amino acids, lipids and redox elements (NADPH) needed to sustain high proliferation rates. Further details are found in the main body of text. PPP—pentose phosphate pathway; IDH1,2/3—Isocitrate dehydrogenase 1,2/3; GLS—glutaminase; GLUD—glutamate dehydrogenase; GLUTs—glucose transporters; MCTs—monocarboxylate transporters; OXPHOS—Oxidative Phosphorylation.

Nutrient addiction is considered a hallmark of cancer and this metabolic reprogramming has two fundamental aspects: first, the rapid production of ATP and intermediates for the synthesis of nucleotides, amino acids, lipids, and redox elements (NADPH); second, the reset of the nutrient-sensing signaling pathways operating in physiological conditions. In addition, to support fast cell division, the tumor cells must generate enough energy and escape from cell cycle checkpoints control. The Warburg effect is a well characterized metabolic phenotype of tumor cells, which consists of increased glycolytic flux for the rapid ATP generation per unit time, instead of producing ATP through oxidative phosphorylation (OXPHOS) [9] (Figure 1). Below, a summary of the main metabolic reprogramming findings on AML is presented.

### 2.1. Glycolysis and Energetic Metabolism

Enhanced glycolysis has been observed in AML cell lines and in human primary AML blasts [10], while both the phosphoinositide 3-kinase (PI3K)/serine-threonine protein kinase B (AKT) and the mammalian target of rapamycin (mTOR) seemingly contribute to this glycolytic metabolism [11,12]. It has also been reported that AML patients have a distinct glucose metabolic signature as well as six serum metabolites involved in the glycolytic pathway which were identified and proposed as new prognostic biomarkers in the subgroup of patients with normal cytogenetics [13].

The high glycolytic profile has been shown to be important for chemotherapy response, having been found to confer greater resistance to apoptosis induced by All-Trans-Retinoic-Acid (ATRA) and arsenic trioxide in primary blasts and AML cell lines, besides having an impact on the clinical prognosis [14]. Increased glycolysis also contributes to the resistance of AML patients to cytarabine [15,16]. The impact on clinical outcome is also reported—AML patients who did not achieve remission when compared to, either, healthy controls, or remission patients, show increased levels in distinct glycolysis elements, such as HIF1a, hexokinase 2 (HK2), glucose transporter 1 (GLUT1), and lactate dehydrogenase (LDH) [15]. Thus, the magnitude of myeloblast glycolysis has been proposed as an effective method to determine the pre-treatment prognosis of AML [10]. Additionally, leukemic stem-cells (LSCs) might have a role in inducing insulin resistance and decreasing insulin secretion, in order to increase the supply of glucose to malignant cells, therefore inducing alterations of systemic physiology by co-opting systemic regulatory mechanisms in their favor [17]. Metabolic plasticity allows AML cells to balance between glycolysis and OXPHOS, however, these metabolic adaptations likely differ between AML sub-types [12] and also within leukemic cells from the same patient [18].

### 2.2. Amino Acid Metabolism

Metabolic reprogramming in cancer cells occurs far beyond the Warburg Effect [19,20]. The high glycolytic rate with concomitant ATP production requires the availability of precursors for anabolic pathways, such as intermediates of the tricarboxylic acid (TCA) cycle and the pentose phosphate pathway (PPP) (Figure 1). Biosynthetic pathways are also required for the production of nutrients and metabolization of the three major classes of macromolecules (proteins, lipids, nucleic acids), essential for cell growth and proliferation. Commonly, other carbon sources such as amino acids, particularly glutamine, allow malignant cells to rely on high glycolytic rates. Glutaminolysis is an important mitochondrial metabolic pathway catalyzed by glutaminase (GLS) (Figure 1). Glutamine enters cells through specific transporters, is catabolized into ammonia and glutamate, which in turn could be deaminated and have its carbon skeleton converted to alfa-ketoglutarate (alpha-KG) to support anaplerosis of the TCA cycle. Alpha-KG can proceed backwards through the TCA cycle, by reductive carboxylation operated by the enzyme isocitrate dehydrogenase (IDH) producing citrate and further supporting synthesis of acetyl-CoA and lipids (Figure 1). The alpha-KG also works on glutamine metabolism and can be oxidatively metabolized via TCA to lactate. Apparently, this process might support lipogenesis in tumor cells [21].

Glutaminolysis supplies nitrogen for protein and nucleotide synthesis and is necessary to maintain the redox state through the production of NADPH (Figure 1). It is reported that glutamine reactivates the mammalian target of rapamycin complex 1 (mTORC1), specifically through its conversion to glutamate and restoration of non-essential amino acid pool [22]. It is also documented that glutamine levels control OXPHOS in AML cells and glutaminolysis inhibitors activate mitochondrial apoptosis, whereas in normal CD34+ hematopoietic progenitors, glutaminase isoform 1 (GLS1) inhibition does not result in reduced proliferation and/or survival of these cells [23,24]. Therefore, inhibiting the glutamine metabolism in AML has been an appealing strategy against this malignancy [25,26]. Nevertheless, the role of glutamine in cancer metabolism is far more complex. Other amino acids such as asparagine have been shown to cooperate with glutamine in favoring malignant cell metabolism. In cells deprived of glutamine, asparagine can rescue the growth and survival of cells, as it maintains protein synthesis (Figure 1) [27]. Accordingly, the introduction of asparaginase blocks the ability of cells to adhere to glutamine deprivation [27]. Human LSCs have been consistently described as selectively dependent upon amino acid metabolism (e.g., glutamine, glutamate, and proline) for OXPHOS and survival in de novo AML patients, while not depending on amino acid metabolism when isolated from relapsed patients [28,29]. LSCs from relapsed patients can, apparently, metabolically compensate by increasing fatty acid metabolism [29].

### 2.3. Fatty Acid Metabolism

Lipid metabolism dysregulation has been established as an important metabolic phenotype in malignant cells. It is overly reported that the various processes inherent to lipid metabolism, such as fatty acid synthesis and fatty acid oxidation (FAO) are altered in many cancers [30,31]. In AML a reprogramming of lipid metabolism has also been shown, both in the leukemic hematopoietic precursors and the signaling that these cells induce on the bone marrow microenvironment, namely on bone marrow adipocytes [32]. Bone marrow adipocytes interact with primary myeloid leukemia cells from patients promoting their survival and proliferation [33]. In this adipocyte-leukemic cell interaction, blasts alter metabolism of adipocytes by activating lipolysis thus allowing the transfer of fatty acids from adipocytes to blasts. Curiously, a reservoir of LSCs was found in gonadal adipose tissue, further documenting the dependence of hematopoietic stem-cells (HSCs) and LSCs on lipid metabolism [34].

FAO, which takes place in the mitochondrial matrix, is a process used by leukemic cells to promote their survival and quiescence [35]. The exploration of this metabolic pathway by the malignant cells allows for both the generation of citrate in the TCA cycle and of antioxidant defenses and reduced coenzymes, FADH_2_ and NADH, which are then oxidized to generate ATP via OXPHOS (Figure 1). Carnitine palmitoyl transferase 1A (CPT1A) and carnitine transporter CT2 (SLC22A16), rate limiting actors of FAO, are overexpressed in AML and constitute novels targets for a subset of AML [36,37] (for a review, see [18]).

The optimization of lipid metabolism is essential for cell proliferation and synthesis of membrane lipid components under stress situations [35]. Of seemingly particular relevance in the global context of metabolism reprogramming is the finding that lipid anabolism is strongly altered in *IDH1* mutant AML cells with a crucial dysregulation of fatty acid metabolism and fluxes [32].

### 2.4. Metabolic Enzymes and Epigenetic Modifiers

In the last decade, mutations in two key metabolic enzymes, cytosolic and mitochondrial isocitrate dehydrogenases (IDH1 and IDH2, respectively), have been identified in myeloid malignancies [38,39]. IDHs catalyze the oxidative decarboxylation of isocitrate producing alpha-KG, NADPH/NADH, and carbon dioxide (Figure 1). These reactions provide the NADPH needed for lipid biogenesis and protection to oxidative damage, facilitating the function of alpha-KG-dependent dioxygenases. Genes encoding isoforms of *IDH1* and *IDH2* are mutated in approximately 15–20% of AML patients [40,41]. These mutations are acquired early in the leukemogenic process and are kept stable throughout the disease’s evolution, the leukemic *IDH* mutated clone being able to survive the initial treatment, thus contributing to relapse [42]. *IDH* mutations induce a neomorphic activity (resulting from a rearrangement of the enzyme’s active site), leading to the production of the oncometabolite 2-hydroxyglutarate (2-HG) (Figure 1), which is a competitive inhibitor of multiple alpha-KG-dependent dioxygenases, including histone demethylases and the TET family of 5-methlycytosine hydroxylases (e.g., TET2). 2-HG promotes DNA and histone hypermethylation, altering the epigenetic landscape of cells. This phenomenon blocks cellular differentiation and promotes cellular transformation and, thus, tumor initiation and progression [38,43]. *IDH* mutations are both mutually exclusive with each other and with mutations in *TET2*. In most studies, *IDH* mutations were found to be heterozygous, resulting in a gain of function phenotype, occurring primarily in three arginine residues critical for isocitrate binding-the R132 codon in *IDH1* and the R140 and R172 in *IDH2* [44,45]. It has been proposed that the mutant allele burden of *IDH* can be a biomarker of response to treatment in AML patients [46]. Nonetheless, there is still some controversy about the prognostic value of *IDH* mutations in AML. A systematic review and a meta-analysis concluded that *IDH1* mutations confer worse OS and event-free survival (EFS), especially in patients with normal cytogenetics, and that *IDH2* mutation improves OS particularly in patients with intermediate-risk AML [47].

### 2.5. Signaling Pathways and Autophagy

As mentioned above, multiple signaling pathways control metabolic reprogramming of leukemic cells including PI3K/AKT, mTOR, and the AMP-activated protein kinase (AMPK). This is a complex network hub of signaling proteins and kinases functioning as sensors and regulators of response to environmental stimuli which govern cell growth, proliferation, and survival. These pathways integrate the availability of nutrients, mitogenic growth factors, cellular energy levels, and stress with the biosynthesis of three major classes of macromolecules (proteins, lipids, and nucleic acids). These signaling pathways have been reported to regulate normal hematopoiesis, reviewed in [48], but are frequently overexpressed in AML [49]. While aberrant PI3K/AKT pathway activation is found in 50%–80% of AML cases [50], mTORC1 appears to be active in all reported AML cases [51,52].

mTORC1 is an important anabolic kinase in the regulation of normal hematopoiesis which is inhibited when amino acids and oxygen levels are decreased. Insofar, mTORC1 suppression contributes to HSCs maintenance by repressing mitochondrial biogenesis. Being HSCs in the hypoxic environment of the bone marrow, they are dependent on glycolysis for their energy needs more than of OXPHOS, so mTORC1 suppression is vital for their homeostasis [53,54]. mTORC1 is negatively regulated by AMPK, a key sensor of the cellular energy status, which suppresses anabolic processes and promotes multiple catabolic processes critical in maintaining metabolic homeostasis and survival. In HSCs, AMPK was demonstrated to be fundamental for the maintenance of energy homeostasis [55]. Our results also showed that AMPK is activated in some AML cell lines, which can be related to their metabolic profile [56], suggesting that the use of metabolic inhibitors which activate AMPK in combination with chemotherapy should be explored in the treatment of AML.

Constitutive activation of mTORC1 has been systematically identified in patients’ myeloid blast cells. There are several mechanisms responsible for this activation, namely, activating mutations in the receptor tyrosine kinase *FLT3*, mutations in the *c-KIT* gene, increased production of angiogenic factors (vascular endothelial growth factor (VEGF) and angiopoietins) and increased expression of *IGF-1* growth factor [57]. Furthermore, activating signals from the microenvironment, including chemokines and adhesion molecules, are also factors that contribute to this signaling pathway activation [58]. mTORC1 is found to be activated even in the absence of an elevated PI3K/AKT signaling [12,59]. In spite of this evidence, the role of AMPK, mTORC1 and/or PI3K/AKT in AML cells is still controversial, having both tumor-suppressor and -promoter functions been assigned to these actors [48,52,56,60,61,62,63,64,65]. Importantly, this signaling network plays a key role in controlling the balance between the promotion of anabolism and the suppression of catabolic pathways such as macroautophagy (hereafter named autophagy). Autophagy is a tightly-regulated catabolic response to numerous cellular stresses such as nutrient starvation, hypoxia, growth factor deprivation, metabolic stress, and most notably, cancer therapy drugs.

Curiously, similar to the signaling pathways controlling this catabolic degradative process, dysregulation of autophagy has been extensively described in AML with both tumor-suppressor and -promoter functions [56,66,67,68]. Autophagy is often found reduced in human AML blasts and the loss of key autophagy genes leads to leukemia initiation and progression in mouse models [69,70,71]. Importantly, different sequencing and in silico studies showed a high frequency of AML patients carrying heterozygous deletions, missense mutations, or copy number variations of autophagy genes, particularly AML patients with complex karyotypes [69,72,73]. These studies are further supported by the correlation between the heterozygous chromosomal loss of 5q, 16q, or 17p and the encoded regions for the autophagy genes *ATG10* and *ATG12*, *GABARAPL2* and *MAP1LC3B*, or *GABARAP*, respectively [69]. Accordingly, the heterozygous loss of *ATG5* increases the progression and aggressiveness of an AML model [69]. A recent study challenges these observations and suggests a tumor-promoting role for autophagy, by showing that knockdown of *ATG5* in primary AML cells resulted in impaired engraftment of human cells in immunodeficient NSG mice [74].

We have previously addressed the relationship between signaling pathways and autophagy in the context of AML cell lines [12]. The results showed that preferential OXPHOS metabolism of AML cells is associated with constitutive co-activation of AMPK and mTORC1 and increased autophagy, while a glycolytic profile is mainly associated with AKT/mTORC1 activation and low autophagy flux, highlighting the relevance of signaling pathways and autophagy on metabolic reprograming of AML [12].

## 3. Modulation of AML Metabolism

New drugs targeting metabolism in AML have been developed and studied in preclinical and clinical trials. These drugs target, among other, glycolysis, amino acids and fatty acid metabolism, signaling pathways, autophagy, metabolic enzymes (e.g., IDH), and epigenetic modifiers. Importantly, a clinical trial, still in the recruitment phase, aims to determine the global metabolic adaptations that occur following exposure to standard chemotherapeutic agents using peripheral blood mononuclear cells from AML patients (NCT02581917). Below, a brief summary of the main approaches targeting metabolism in AML is presented (data from https://clinicaltrials.gov).

### 3.1. Targeting Glycolysis and Energetic Metabolism

An effective anti-leukemia strategy is to counteract the up-regulated glycolysis, following the example of its proven application in several neoplasms. Glycolysis can be blocked by substrate restriction and/or by the inhibition of glycolytic enzymes. Reducing glucose uptake by attacking one of the two main transporters, such as GLUT1, is a strategy that has also been explored in hematological malignancies, such as acute lymphoid leukemia and multiple myeloma [75,76]. In AML, there is also evidence that high levels of GLUT1 are associated with poor response to chemotherapy and inhibiting glycolysis is a potential anti-tumor strategy [16]. Inhibition of glycolysis with glycolytic inhibitor 2-deoxyglucose (2-DG), which cannot be metabolized by glycolytic enzymes, has been documented to potentiate the cytotoxicity of the chemotherapeutic drug cytarabine [13]. It was also found that 2-DG has anti-leukemic activity in AML cell lines with *FLT3* and *c-KIT* mutations, but this warrants validation in a clinical setting [77]. Another potent inhibitor of glycolysis is 3-bromopyruvate (3-BrPA), which directly inhibits HK2 and triggers apoptosis in a variety of cancers, including the AML cell line HL-60 [16]. It is also reported that AML cell lines resistant to sorafenib (a kinase inhibitor used for FLT3-ITD positive AML) are sensitive to both 2-DG and 3-BrPA [78]. Nevertheless, inhibiting glycolysis requires further investigation to define the clinical applicability of this strategy. Regarding oxidative metabolism, there is an ongoing phase I clinical trial (NCT02882321, Table 1) using IACS-010759, a potent electron transport chain complex 1 inhibitor. This drug has shown cytotoxic efficacy by inducing AMPK-dependent apoptosis in OXPHOS-reliant AML, likely owing to a combination of energy depletion and reduced aspartate production that leads to impaired nucleotide biosynthesis [79].

### 3.2. Targeting Amino Acid Metabolism

Glutamine dependence is a common metabolic strategy in many neoplasms, therefore constituting a potential metabolic target to be explored [83]. Recently, it was reported that depleting glutamine in culture media or decreasing its catabolism trough GLS1 inhibition induced significant growth suppression and cell death in AML cell lines [84]. Furthermore, the knockdown of the GLS1 and its inhibition with CB-839 reduces OXPHOS, inhibits leukemic cell proliferation and induces apoptosis without affecting normal CD34+ hematopoietic cells [84]. It was also shown that glutaminolysis inhibition triggers mitochondria-dependent cell death and sensitizes AML cells to the BCL-2 inhibitor, ABT-199 [80]. This combination is an example of synthetic lethality that has been explored in many tumors by associating effective and less toxic drugs [24,85]. Another example is the combination of glutaminase inhibitor CB-839 with the tyrosine kinase inhibitor (AC220-Quizartinib), which causes major loss of viability through apoptotic cell death in *FLT3*-mutated AML cell lines and improves the survival in a patient-derived xenograft AML mouse model [86]. A clinical trial that is now completed used a combination of CB-839 with azacitidine, a DNA methyltransferase (DNMT) inhibitor, which has in vitro and in vivo demethylating effects (NCT02071927, Table 1). Although no results were posted so far, this same combination has been proven effective in up to 60% of patients with myelodysplastic syndromes in a Phase III randomized controlled trial [87].

As mentioned above, asparagine is also a key player in the metabolism of malignant cells and can be explored in therapeutic approaches. Since asparaginase is already approved for treating acute lymphoid leukemia in children, it has been included in several clinical trials in AML patients integrating strategies that contemplate the complex metabolism of asparagine and glutamine, or in combination with low doses of cytarabine (NCT02283190 and NCT01810705, Table 1) [88].

Preclinical studies have demonstrated that AML blasts are dependent on arginine to proliferate and survive since, at diagnosis, most leukemic cells have deficiencies in the arginine-recycling pathway enzymes [89]. BCT-100, a pegylated recombinant arginase, causes a depletion of intra- and extra-cellular arginine, resulting in halted proliferation of blasts and a reduction of engraftment in vivo [89]. Depletion of arginine is, therefore, an important strategy of ongoing clinical trials (NCT02899286 and NCT01910012, Table 1). Overall, there is an emerging interest in targeting amino acid metabolism, given its crucial role in sustaining the growth and proliferation of malignant cells, including AML ones.

### 3.3. Targeting Fatty Acid Metabolism

Both increased lipogenesis and FAO allow malignant cells to survive periods of metabolic stress [30]. Some studies assessed the therapeutic potential of counteracting alterations of lipid metabolism in AML [90]. For example, the drug ST-1326, an inhibitor of CPT1A crucial for the acyl-CoA shuttling to mitochondria, slows the proliferation of leukemic cells and results in their apoptosis, sensitizing these malignant cells to the cytotoxic effect of cytarabine [91]. Interestingly, a small molecule with broad anti-tumor activity (SR9243) was described as simultaneously reducing the intracellular concentration of glycolytic metabolites and preferentially suppressing lipogenesis in malignant cells [92]. Conversely, FAO antagonizes the oligomerization of the pro-apoptotic components BAX and BAK, thus compromising cell death and raising the threshold of response to pro-apoptotic stimuli [93]. Furthermore, the FAO inhibitor Avocatin B was found to decrease NADPH levels and cause reactive oxygen species (ROS)-dependent leukemic cell death in AML [94]. FAO is, therefore, important for AML and its inhibition could become another potential target of cancer therapy. However, given the complexity of lipid metabolism and its signaling network in cancer cells, it is still difficult to select the best lipid dysregulation-targeting therapy.

### 3.4. Targeting Metabolic Enzymes and Epigenetic Modifiers

There are several clinical trials featuring drugs that inhibit mutated *IDHs* (Table 1) with promising results and low toxicity profiles in both refractory/resistant leukemia patients and in untreated patients [95]. Enasidenib (AG-221) is a covalent inhibitor of R140Q- and R172K-mutated *IDH2* which does not affect the activity of wild-type *IDH2* [96]. In preclinical studies, enasidenib inhibited the aberrant IDH2 protein, decreased total serum 2-HG levels in more than 90%, reduced abnormal histone hypermethylation, and restored myeloid differentiation [97]. Previous clinical trials with enasidenib in relapsed or refractory AML patients provided positive results [98]. Currently, enasidenib is being tested in a multicenter randomized phase III study (NCT02577406, Table 1). This drug is well tolerated, but almost 12% of the patients experienced a differentiation syndrome similar to the one seen with ATRA therapy in acute promyelocytic leukemia patients [99].

Ivosidenib (AG-120), IDH305, AG-881, BAY-1436032, and FT-2102 are some of the IDH1 inhibitors under investigation (Table 1). Ivosidenib is being used in different ongoing clinical trials, and both the patient response rates and differentiation syndrome (associated with the terminal differentiation of the leukemic clone) are similar to enasidenib’s [100]. A small number of patients with complete remission had clearance of the mutated clone and remained in the trial for over a year [95]. In a recent study of ivosidenib monotherapy in advanced *IDH1* mutated relapsed or refractory AML, similar results were obtained with respect to the duration of remission, molecular remission, and therapeutic-related adverse events [101]. In conclusion, these drugs are profiled as optimal agents for use in combination strategies, since they act against specific targets and exhibit low extra-medullary toxicity.

Mutations in *IDH1/2* and in the epigenetic modifier enzymes—*DNMT3A* and *TET2*—were detected in more than 70% of AML patients [45]. All of these mutations are associated with epigenetic aberrations in the pathogenesis of myeloblastic leukemia, so it is necessary to point out, albeit briefly, the clinical experience acquired with drugs that act on DNA methylation. Decitabine and 5-azacitidine—hypomethylating agents—are standard drugs used in myelodysplastic neoplasms and some AML subtypes with proven clinical efficacy and acceptable toxicity (NCT01757535, Table 1). Several multicenter and randomized trials showed that they increase OS when compared to conventional options, including low-dose-cytosine arabinoside and intensive chemotherapy [102,103]. It was also found that decitabine was effective even in patients with high-risk karyotype and/or *TP53* mutations, constituting a possible therapeutic option in cases with poor prognoses [104]. In fact, these drugs altered the treatment algorithm of elderly patients, since they have the capacity to prolong patient survival, keeping the disease stable and/or improving cytopenia, even if remission is not achieved [105].

Guadecitabine is a new-generation hypomethylating agent with improved uptake of decitabine, the active metabolite, into the DNA of rapidly dividing cancer cells [106]. Decitabine restores expression of silenced tumor suppressor genes and tumor-associated antigens and may sensitize tumor cells to other anticancer agents, including immunotherapeutic drugs. Guadecitabine is being used in clinical trials (NCT02920008, Table 1) yielding good therapeutic responses, a pattern that overlapped with other hypomethylating agents [107]. However, in hematologic malignancies, such agents have a double effect on the immune system: they increase anti-tumor immune response and, conversely, dampen the immune response by increasing the expression of immune-checkpoint molecules. Clinical trials exploring the combination of hypomethylating agents (e.g., azacitidine) with immune-checkpoint inhibitor drugs (e.g., nivolumab) are underway, with promising results (NCT02397720, Table 1) [82].

An important therapeutic strategy in AML is the combination of venetoclax (a BH3-mimetic that blocks the anti-apoptotic BCL-2, frequently overexpressed in AML, reviewed in [108]) with low-doses of cytarabine or decitabine or azacitidine in previously untreated patients older than 65 years [109]. Recently, it was shown that LSCs from patients undergoing treatment with venetoclax and azacitidine displayed decreased levels of alpha-KG and increased levels of succinate, pointing at an alteration of carbon flux in the TCA cycle, thus suggesting an efficient and targeted suppression of OXPHOS [110]. It was also found that *IDH1*- and *IDH2*-mutant primary human AML cells were more sensitive than *IDH1/2* wild-type cells to venetoclax, which was due to mitochondrial electron transport chain alterations that lowered the threshold of apoptosis induced by this BCL-2 antagonist [111]. In view of these promising assays, biomarkers are being studied that predict the efficacy of venetoclax in each patient [112].

### 3.5. Targeting Signaling Pathways and Autophagy

Due to the relevance of PI3K/AKT, mTOR, and AMPK on AML pathogenesis, these signaling pathways present appealing therapeutic targets. In a murine model in which primary leukemic cells were co-cultured with stroma, PP242, a mTOR inhibitor, simultaneously showed inhibitory activity against mTORC1 and mTORC2 as well as an antagonism against the stromal-induced survival signal, by suppressing expression of CXC chemokine receptor type 4 (CXCR4) [113]. This effect on the medullary microenvironment should be emphasized, since this signaling affects the stemness of leukemic precursors and contributes to chemoresistance of leukemic cells. Although the first mTOR inhibitors (rapamycin and analogues) had a cytostatic effect and a low inhibitory growth effect, other inhibitors subsequently demonstrated synergistic action with either standard chemotherapy, or with other mTOR inhibitors [65,114]. In preclinical models, several dual mTORC1/2 inhibitors and dual PI3K/mTOR inhibitors showed greater anti-leukemia activity [115,116]. The interactions between mTORC1 and PI3K/AKT are complex, with mTORC1 exerting a negative control of PI3K/AKT that is alleviated upon mTORC1 inhibition by sirolimus [117].

A potential mechanism for PI3K activation in AML might implicate increased VEGF levels [48]. Enhanced VEGF signaling has been proposed as a cause of reduced apoptosis susceptibility upon AML treatment [118]. Recently, it was shown that blocking VEGF receptor 2 (VEGFR2) signaling sensitized chemoAML (human AML cells from terminally ill mice treated with chemotherapy) to chemotherapy by inducing a transcriptional program that promotes mitochondria biogenesis associated with increased oxidative stress, further suggesting mitochondria function is a vulnerable target for chemotherapy in AML [119].

AMPK pathway has also been implicated in hematological malignancies, mainly as a tumor suppressor axis [63,120,121], by providing metabolic stress resistance to leukemia-initiating cells. The anticancer properties of metformin were first associated with a complex mechanism of AMPK activation, but recently different studies have demonstrated that antitumor effects of metformin— and, globally biguanides—are AMPK-independent, reviewed in [59]. Notwithstanding this controversy in the context of hematologic malignancies, metformin was reported to activate AMPK and inhibit growth of AML cell lines and primary AML cells, while sparing normal hematopoiesis ex vivo [63]. However, additional studies in AML cell lines showed that metformin mediated effects on apoptosis, and proliferation is preserved even in cells with siRNA-downregulated AMPK, thereby suggesting AMPK-independent effects [122]. Among the direct activators of AMPK, GSK621 was reported to selectively kill AML cell lines and primary cells without affecting normal hematopoietic progenitors [62]. Nevertheless, our previous results have shown that AMPK is constitutively activated in some AML cell lines and that standard chemotherapy targets AMPK for degradation [12,56]. These somehow contradictory results suggest that further studies should be performed to clarify the value of AMPK as a therapeutic target.

Although several studies have demonstrated beneficial effects of drugs targeting signaling pathways in preclinical models of AML, results of clinical trials are either modest, or disappointing (reviewed in [115,123,124]). Table 1 illustrates some of the clinical trials targeting mTOR (NCT01154439 and NCT01869114) or PI3K/AKT (NCT01396499 and NCT02392572) signaling pathways.

Decreased mTOR activity as well as nutrient or growth factor deprivation increase autophagy, therefore constituting an essential survival pathway [125]. Autophagy is critical for the maintenance of HSCs, but plays context-dependent roles in leukemia initiation, progression, and drug resistance [126]. Autophagy is needed for the maintenance of murine HSCs and its impairment diverts normal HSCs to a pre-leukemic state [67]. These findings are supported by evidence showing that hematopoietic-specific deletions of *ATG5*, *ATG7,* or the *ULK1*-interacting partner Fip200 diminished normal HSCs activities, promoted a pre-leukemic phenotype and, consequently, impaired survival of these mice [66,69,127]. Other studies showed that *ATG5* or *ATG7* are required for the efficient initiation of AML associated with *MLL* (mixed lineage leukemia)-*AF9* fusion gene, while autophagy is no longer required for the maintenance of established AML or LSCs functions in secondary xenotransplantation experiments [128,129,130,131]. In contrast, knockout of *ATG5* or *ATG7* in a murine *MLL-ENL* (eleven nineteen leukemia) AML model decreased the number of functional LSCs, increased activation of mitochondria and ROS levels in these cells, and extended survival of leukemic mice [132]. These aspects were elegantly reviewed in [126].

Recently, it was also shown that autophagy suppression mediated by receptor tyrosine kinase effectors-mTORC1 signaling stabilize mutant FLT3 in AML, while an increase in autophagy was achieved through receptor tyrosine kinase inhibition and led to FLT3 depletion [71]. In contrast, a different study showed that FLT3-ITD increases autophagy in AML cell lines and AML patient blasts, and that inhibition of autophagy abolishes FLT3 inhibitor resistance [133]. Recent studies also revealed inconsistent levels of basal autophagic flux in various leukemic cell lines [12,56,74] and purified CD34+ cells from AML patients [74]. Particularly high autophagic levels were found in poor risk AML, which are frequently associated with *TP53* mutations [74].

Altogether, these contradictory data suggest a highly complex, context-dependent role for autophagy in leukemic transformation and LSCs properties in AML [126]. To further complicate this scenario, autophagy is also established as one of the resistance mechanisms of leukemic cells to chemotherapy [56,67]. However, although autophagy is generally considered a critical target in the therapeutic approach to myeloblastic leukemia, it is documented that the effectiveness of this proteolytic system will depend on the subtype of AML [12,134]. Clinical trials with hydroxychloroquine, an inhibitor of autophagy, showed increased cytotoxicity of conventional chemotherapy in leukemic cells (NCT02631252, Table 1) [135,136]. Although there are still diverging findings on this subject, it is accepted that autophagy has a versatile role which depends, simultaneously, on both the progenitor and driver engaged in leukemia’s transformation process, as well as the state of leukemic expansion.

## 4. Conclusions

AML is a very heterogeneous and complex group of diseases and, despite advances in therapeutic strategies, there is still a high rate of resistance to the standard treatment. Accordingly, it is necessary to find innovative ways of approaching and treating patients with AML. Age, performance status, white blood cell count, secondary disease, cytogenetic risk, and the mutational state are incorporated in the predictive models of resistance [137]. However, other variants such as the co-occurrence of mutations, the minimal residual disease, and the emergence of sub-clones with a different sensitivity to chemotherapy are also key players in the search for a more effective therapy [5,138]. In this context, it is tempting to propose that the patient’s metabolic profile, as well as the metabolic phenotype of malignant cells, may be considered prognostic factors in AML. The modulation of metabolic reprogramming and the use of directed drugs open new promising possibilities in fighting AML. Nevertheless, results supporting this hypothesis are mainly based on preclinical and in vitro studies—not on data from clinical trials, which are still scarce. Another aspect to consider on metabolic targeting is that these approaches will likely have a differential effect on normal hematopoiesis [139].

The results reviewed above show that energy metabolism is a fundamental process that should be considered in AML therapy. Depending on the balance between glycolysis and OXPHOS, the amino acid requirements of AML cells also change, and this could be useful for metabolic targeting. Importantly, lipid metabolism is emerging with great therapeutic potential given the alterations found in AML cells. Nevertheless, the knowledge of the complexities of lipid metabolism is still scarce, and probably this is one of the reasons why no current clinical trials are found which feature drugs targeting this axis of metabolic reprogramming. Summing up, more preclinical and clinical studies have to be performed in order to establish the best metabolic target (or combination of targets) that is better suited to affect LSCs sparing HSCs. Hopefully, the current preclinical and clinical ongoing studies will shed light on the yet unknown aspects of metabolic reprogramming in AML.

In this review, we intended to provide an updated look at AML therapy; this new perspective will, eventually, allow age constraints that current therapies still have to be overcome and, in addition, facilitate the prediction of better results in refractory cases in the near future.

## Figures and Tables

**Table 1:** Modulators of metabolism and clinical trials on acute myeloid leukemia.

Drugs	Drugs in Combination	Clinical Trial	Status
**Targeting Oxidative Phosphorylation (OXPHOS)**			
IACS-010759		NCT02882321 Phase I study to dose escalation and dose expansion of the IACS-010759.	Active, recruiting [79]
**Targeting amino acid metabolism**			
CB-839	Azacitidine	NCT02071927 Phase I/II study to evaluate the dependence of AML and acute promyelocytic leukemia on glutamine.	Completed, no results posted [80,81]
Erwinase (ASPARAGINASE)		NCT02283190 Phase I study to determine and analyze the effects of Erwinaze/Asparaginase on reducing serum glutamine levels.	Completed, no results posted
GRASPA (L-asparaginase encapsulated in red blood cells)	Low-dose cytarabine	NCT01810705 Phase II study to evaluate the efficacy and tolerability of GRASPA plus low-dose cytarabine in treatment of AML patients over 65 years old, unfit for intensive chemotherapy.	Completed, no results posted
PEG-BCT-100 (PEGylated recombinant human arginase)		NCT02899286 Phase II, non-randomized, open-label study to evaluate the efficacy of single agent PEG-BCT-100 on arginine depletion in adult patients with relapsed/refractory AML.	Active, not recruiting
ADI-PEG 20 (arginine deiminase formulated with polyethlene glycol)		NCT01910012 Phase II study to evaluate the response rate of arginine depletion by arginine deiminase.	Active, not recruiting
**Targeting metabolic enzymes and epigenetic modifiers**			
Enasidenib (AG-221)		NCT02577406 Phase III study comparing the efficacy and safety of AG-221 versus conventional care regimens in subjects 60 years or older with AML refractory to or relapsed after second- or third-line AML therapy and positive for *IDH2* mutation.	Active, recruiting
Ivosidenib (AG-120)	Azacitidine	NCT03173248 Phase III study to evaluate the efficacy and safety of AG-120 plus azacitidine in adult subjects with previously untreated IDH1m AML who are considered appropriate candidates for non-intensive therapy.	Active, recruiting
IDH305		NCT02381886 Phase I study to estimate the maximum tolerated dose/recommended dose for expansion of IDH305 in patients with advanced malignancies that harbor *IDH1* R132 mutations.	Active, not recruiting
BAY-1436032		NCT03127735 Phase II study to determine the maximum tolerated and/or recommended dose of BAY1436032, a mIDH1 inhibitor in patients with mIDH1-R132X advanced AML.	Completed, no results posted
AG-881		NCT02492737 Phase I study to evaluate the safety, pharmacokinetics, pharmacodynamics, and clinical activity of AG-881 in advanced hematologic malignancies that harbor an *IDH1* and/or *IDH2* mutation.	Completed, no results posted
FT-2102	Azacitidine or cytarabine	NCT02719574 Phase I/II study to evaluate the safety efficacy, pharmacokinetics and pharmacodynamics of FT-2102 as a single agent or in combination with azacitidine or cytarabine.	Active, recruiting
Guadecitabine		NCT02920008 Phase III study of the efficacy and safety of guadecitabine in adults with previously treated AML.	Active, not recruiting
Oral azacitidine	Best-supportive care	NCT01757535 Phase III study to compare efficacy and safety of oral azacitidine plus best-supportive care versus best supportive care as maintenance therapy in subjects with AML in complete remission.	Active, not recruiting
Azacitidine	Nivolumab	NCT02397720 Phase II study to determine the side effects and the best dose of nivolumab and azacitidine with or without ipilimumab for treating patients with AML that have not responded to previous treatment or have returned after a period of improvement or are newly diagnosed.	Recruiting [82]
**Signaling pathways and autophagy inhibition**			
BKM120		NCT01396499 Phase I study to determine the highest tolerable dose and the safety of BKM120, a PI3K kinase inhibitor that can be given to patients with relapsed or refractory leukemia.	Completed, no results posted
ONC201	Low-dose cytarabine	NCT02392572 Phase I/II study to find the highest tolerable dose and safety of ONC201, an inhibitor of the serine/threonine protein kinase AKT and extracellular signal-regulated kinase (ERK), alone or in combination with low dose cytarabine in patients with relapsed or refractory AML, acute promyelocytic leukemia, or myelodysplastic syndromes.	Active, recruiting
Everolimus	MICE-regimen followed by consolidation therapy with idarubicin, cytarabine, and etoposide	NCT01154439 Phase I study to determine the maximum-tolerated dose of everolimus, a derivative of rapamycin that inhibits mTOR, in combination with standard remission-induction therapy comprising mitoxantrone hydrochloride, cytarabine, and etoposide (MICE-regimen) followed by consolidation therapy comprising idarubicin, cytarabine, and etoposide in older patients with newly diagnosed AML.	Active, not recruiting
Sirolimus	Azacitidine	NCT01869114 Phase II trial studies how well sirolimus and azacitidine works in treating patients with high-risk myelodysplastic syndrome or recurrent AML.	Recruiting
Hydroxychloroquine (HCQ)	Mitoxantrone and Etoposide	NCT02631252 Phase I study to find the safest and most effective dose of hydroxychloroquine, an autophagy inhibitor, when combined with the usual medication for AML, mitoxantrone, and etoposide.	Terminated, no results posted
